# 18β-Glycyrrhetinic Acid Inhibits Osteoclastogenesis *In Vivo* and *In Vitro* by Blocking RANKL-Mediated RANK–TRAF6 Interactions and NF-κB and MAPK Signaling Pathways

**DOI:** 10.3389/fphar.2018.00647

**Published:** 2018-06-20

**Authors:** Xiao Chen, Xin Zhi, Zhifeng Yin, Xiaoqun Li, Longjuan Qin, Zili Qiu, Jiacan Su

**Affiliations:** ^1^Department of Orthopedics Trauma, Shanghai Changhai Hospital, Second Military Medical University, Shanghai, China; ^2^China-South Korea Bioengineering Center, Shanghai, China; ^3^Graduate Management Unit, Shanghai Changhai Hospital, Second Military Medical University, Shanghai, China; ^4^Department of Orthopedics, Shanghai Zhongye Hospital, Shanghai, China; ^5^Orthopedic Basic and Translational Research Center, Jiangyin, China; ^6^Jinling High School, Nanjing, China

**Keywords:** 18β-glycyrrhetinic acid, osteoclastogenesis, RANKL, TRAF6, MAPK signaling pathway

## Abstract

Bone metabolism is determined by a delicate balance between bone resorption by osteoclasts and bone formation by osteoblasts. The imbalance due to over-activated osteoclasts plays an important role in various diseases. Activation of NF-κB and MAPK signaling pathways by receptor activator of nuclear factor -κB ligand (RANKL) is vital for osteoclastogenesis. Here, we for the first time explored the effects of 18β-glycyrrhetinic acid (18β-GA), a pentacyclic triterpenoid found in the *Glycyrrhiza glabra* L roots, on RANKL-induced osteoclastogenesis, osteoclast functions and signaling pathways *in vitro* and *in vivo*. In bone marrow monocytes (BMMs) and RAW264.7 cells, 18β-GA inhibited osteoclastogenesis, decreased expression of TRAP, cathepsin K, CTR and MMP-9, blocked actin ring formation and compromised osteoclasts functions in a dose-dependent manner at an early stage with minimal effects on osteogenic and adipogenic differentiation of bone marrow mesenchymal stem cells (BMSCs). For underlying molecular mechanisms, 18β-GA inhibited RANKL-induced phosphorylation of p65, p50, and IκB, blocked p65 nuclear translocation and decreased the DNA-binding activity of NF-κB. Besides, 18β-GA inhibited the activation of the MAPK pathways. Co-immunoprecipitation showed that 18β-GA treatment blocked RANK–TRAF6 association at an upstream site. *In vivo*, 18β-GA treatment inhibited ovariectomy-induced osteoclastogenesis and reduced bone loss in mice. Overall, our results demonstrated that 18β-GA inhibited RANKL-induced osteoclastogenesis by inhibiting RANK expression in preosteoclasts and blocking the binding of RANK and TRAF6 which lead to the inhibition of NF-κB and MAPK signaling pathways. 18β-GA is a promising novel candidate in the treatment of osteoclast-related diseases such as postmenopausal osteoporosis.

## Introduction

Homeostasis of bone metabolism is determined by a dynamic balance between osteoclasts and osteoblasts (Chen et al., 2017b). Disruption of this balance with a higher rate of bone resorption over formation after osteoclast overactivation leads to pathological bone loss in various bone diseases including postmenopausal osteoporosis (PMOP) (Noel et al., 2017), rheumatoid arthritis (RA), and Paget’s disease. Inhibiting osteoclast overactivation is therefore an important treatment strategy for pathological bone loss disease (Swart et al., 2016).

Osteoporosis is the most common osteoclastogenesis-related disease characterized by bone loss and bone tissue microstructural damage, leading to increased bone fragility and the risk of bone fractures (Chandra et al., 2017). The morbidity of osteoporosis correlates with age and sex (25.41% of females vs. 15.33% of males), and PMOP is the most common form of osteoporosis (Majtan et al., 2017). Recently there has been a significant increase in PMOP [14.94% (2005–2008) vs. 27.96% (2012–2015)] accompanied by a significantly increased morbidity among patients over 60 years of age (Chen et al., 2016; Jing et al., 2017).

Osteoclasts derive from monocyte-macrophage lineage and play essential roles in development and various diseases (Teitelbaum, 2000). Osteoclastogenesis *in vivo* is controlled by various cytokines and signaling pathways (Fernandez-Rebollo et al., 2017). The macrophage colony-stimulating factor (M-CSF), receptor activator NF-κB ligand (RANKL) are the two most important factors for osteoclastogenesis both *in vitro* and *in vivo*. M-CSF promotes osteoclast precursors proliferation and survival, increases RANK expression, a prerequisite for osteoclast differentiation (Arai et al., 1999). When RANKL combines with RANK on preosteoclasts, TNF receptor-associated factor (TRAF) 2, 3, 5, and 6 are recruited, which leads to activation of various signaling pathways including the MAPK and NF-κB pathways (Kanzaki et al., 2017). NF-κB activation occurs at very early stage and abrogation of both p50 and p52 causes osteoclastogenesis impairment with an osteoporotic phenotype (Vaira et al., 2008). Activation of these pathways facilitates translocation and activation of the nuclear factor of activated T-cells 1 (NFATc1), the master transcription factor for osteoclastogenesis. It can directly regulate a number of osteoclast-related genes, including tartrate-resistant acid phosphatase (TRAP), calcitonin receptor (CTR), matrix metallopeptidase 9 (MMP-9), and so on (Boyle et al., 2003). Therefore, to target modulation of RANKL-induced signaling pathways to regulate NFATc1 expression could be helpful in the treatment of osteoclast-related diseases like postmenopausal osteoporosis (Chen et al., 2017a,b; Liu et al., 2018; Xin et al., 2018).

18β-Glycyrrhetinic acid (18β-GA) is a pentacyclic triterpenoid derivative of the β-amyrin type produced from the hydrolysis of glycyrrhizic acid (Kiratipaiboon et al., 2015), which is found in the traditional Chinese herb, liquorice. Traditionally, liquorice has been widely used to strengthen the Qi, release cramps, alleviate pain, clear heat and reduce fire toxins in traditional Chinese Medicine. It has been proven to be effective in the treatment of acute liver injury (Yang et al., 2017), hepatocellular carcinoma (Zhu et al., 2017), and colorectal cancer (Wang et al., 2017) as an anti-inflammation agent (Li et al., 2017). This study for the first time characterized the effects of 18β-GA on ovariectomy-induced bone loss *in vivo* and osteoclastogenesis *in vitro*, and explored the molecular mechanisms involved in these processes.

## Materials and Methods

### Reagents

**FIGURE 1:**
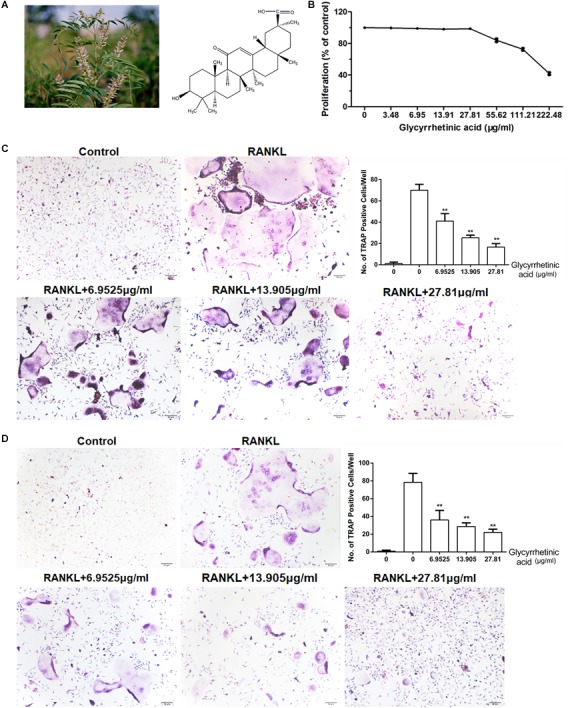
18β-GA inhibits osteoclastogenesis *in vitro*. All the experiments were performed six times and the average was taken. **(A)** Chemical structure of 18β-GA. **(B)** MTT analysis of 18β-GA cytotoxicity in BMSCs. **(C)** Formation of TRAP-positive cells from BMMs and quantification of osteoclast. **(D)** Formation of TRAP-positive cells from RAW264.7 cells and quantification of osteoclast (^∗∗^*P* < 0.01).

18β-GA (**Figure 1A**) was supplied by Nuodande Standard Technical Services (Shanghai, China). It was dissolved in phosphate-buffered saline (PBS) as a vehicle and stored at 4°C until further use. Fetal bovine serum (FBS), penicillin, streptomycin, and all antibodies were purchased from BioTNT (Shanghai, China).

### 3-(4,5-Dimethylthiazol-2-yl)-2,5-Diphenyltetrazolium Bromide (MTT) Assay

We performed an MTT (Sigma-Aldrich, St. Louis, MO, United States) assay following the manufacturer’s instructions to detect 18β-GA cytotoxicity. Bone marrow monocytes (BMMs) were seeded onto a 96-well plate at a concentration of 10 × 10^4^ cells/mL, followed by a 24-h incubation. Doses of 18β-GA at concentrations of 3.48, 6.95, 13.91, 27.81, 55.62, 111.21, and 222.48 μg/ml were added to the wells and cultured with the cells for 72 h. The concentration of MTT solution was 50 μg/μL, and the absorbance at 490 nm was measured by an ELISA plate reader.

### *In Vitro* Osteoclastogenesis Assay

Bone marrow monocytes were isolated from the femoral cavity of C57/BL6 mice (Qiu et al., 2007). Briefly, BMMs were cultured with Dulbecco’s Modified Eagle low glucose medium with added FBS and antibiotic mixtures. After 72 h, the cell medium with non-adherent cells was removed. The remaining adherent colonies were cultured for another 2 weeks and passaged and subcultured. The cultures of the third generation BMMs were added to 48-well plates (2.0 × 10^4^ cells/well). One group of plates was the control group, while four other groups treated with 18β-GA (0, 6.9535, 13.905, and 27.81 μg/ml), 50 ng/mL of M-CSF (macrophage colony-stimulating factor), and 50 ng/mL of RANKL were used to stimulate the 18β-GA-treated cells. After 5 days, the cells showed osteoclast activity according to tartrate resistant acid phosphatase (TRAP) staining (Sigma-Aldrich, St. Louis, MO, United States). The cells with >5 nuclei were counted as osteoclasts. RAW264.7 cells were obtained from the cell bank of the Chinese Academy of Sciences (Shanghai, China), and treated as described above.

An actin ring formation study was performed as previously described (Koide et al., 2009). Briefly, the cells were cultured for 48 h in 96-well plates (1.0 × 10^4^ cells/well), fixed with formaldehyde, permeabilized with Triton-X 100, and cultured with rhodamine-conjugated phalloidin to visualize F-actin. The average of three repeated experiments was used in the analyses. All the experiments were carried out six times.

### *In Vitro* Osteogenesis Assay and Adipogenesis Assay

The effects of 18β-GA on osteoblast differentiation and bone nodule formation. Bone marrow mesenchymal stem cells (BMSCs) were isolated from 4-week-old C57BL/6 by flushing femurs and tibias. To induce osteoblastic differentiation, confluent MSCs were cultured in complete medium supplemented with 10^-8^mol/L dexamethasone, 10 mM β-glycerophosphate and 50 μg/ml ascorbic acid. Differentiated cells were stained with alkaline phosphatase (ALP) staining and alizarin red staining at indicated day. To induce adipogenic differentiation, confluent MSCs were cultured in complete medium supplemented with 50 mg/L Vitamin C, 10 mmol/L β-sodium glycerophosphate and 10^-8^mol/L dexamethasone. Cells were stained with Oil red O staining.

### Pit Formation Assay

A pit formation assay was performed as previously described (Takatsuna et al., 2005) to determine the effect of 18β-GA treatment on bone resorption. Briefly, after mature osteoclasts were observed, osteoclasts were digested by collagenase and seeded (3.0 × 10^3^ cells/well) onto a bone biomimetic synthetic surface (OsteoAssay Surface 24-multiple well plates; Corning, Corning, NY, United States) plate with 50 ng/mL of M-CSF and 50 ng/mL of RANKL. The cells were treated with or without 18β-GA (0, 6.9535, 13.905, and 27.81 μg/ml) for an additional 2 days. Two days later, the surfaces were cleared with sodium hypochlorite (0.5%) and PBS followed by air drying for 2–3 h. The osteoclast resorbing area was observed using a light microscope and calculated using Image Pro software (Media Cybernetics, Rockville, MD, United States).

### Immunofluorescence Staining

Immunofluorescence was used to evaluate whether 18β-GA treatment affected the nuclear translocation of p65 (Koide et al., 2009). Briefly, BMMs were fixed with paraformaldehyde, followed by washing with Triton X-100, then incubated with monoclonal anti-p65 antibody, multiclonal goat anti-mouse IgG antibody, and streptavidin conjugated with fluorescein.

### Electrophoretic Mobility Shift Assay (EMSA)

The quantity of DNA binding protein was measured using an EMSA. Briefly, the BMMs were treated with RANKL or RANKL/18β-GA (0 and 27.81 μg/ml), then washed three times using PBS. All adherent cells were then scraped off the substratum and centrifuged. A ^32^P-labeled DNA duplex was added to the suspension and incubated in a water bath. The mixture was briefly centrifuged before being resolved using sodium dodecyl sulfate-polyacrylamide gel electrophoresis (SDS-PAGE). The gel was run at 4°C for 1–4 h, followed by autoradiography.

### Western Blotting

The influence of 18β-GA treatment on the NF-κB and MAPK pathways and the expression of osteoclastogenesis-related markers were determined using western blot analysis. Briefly, western blot analysis was performed on cells treated with or without 18β-GA for 0, 15, 30, 45, and 60 min to evaluate phosphorylation of IκB, p65, p50, ERK, JNK, and p38. The control group and other groups stimulated by RANKL with or without 18β-GA (0 and 27.81 μg/ml) were cultured in 96-well plates (1 × 10^4^ cells/well) for 5 days. The expression levels of CTR, cathepsin K, TRAP and MMP-9 were then evaluated. The total protein was extracted from the cells using M-PER mammalian protein extraction reagent (Pierce, Rockford, IL, United States). Equal amounts of protein (10 μg per lane) estimated by a bicinchoninic acid (BCA) protein assay kit (Pierce) were loaded onto (11%) SDS-PAGE gels and transferred onto nitrocellulose membranes. The blots were probed with a monoclonal antibody against human anti-TRAP (41-Q) (1:350), anti-Cathepsin K (E-7) (1:500), anti-MMP-9 (7-11C) (1:400), anti-CTR (N-20) (1:200), anti-p65 (A-12) (1:350), anti-P-p65 (A-8) (1:500), anti-p50 (D-6) (1:250), anti-P-p50 (A-8) (1:400), anti-IκBa (H-4) (1:350), anti-P-IκBa (B-9) (1:500), anti-ERK (C-9) (1:200), anti-P-ERK (12D4) (1:400), anti-JNK (D-2) (1:500), anti-P-JNK (G-7) (1:400), anti-p38 (F-9) (1:300), anti-P-p38 (D-8) (1:300), and anti-beta actin (C-2) (1:1000) (Santa Cruz, Dallas, TX, United States), followed by the secondary HRP conjugated anti-mouse/rabbit antibody (Santa Cruz). After washing, the bands were detected by chemiluminescence and imaged with X-ray films. Beta actin was used as an endogenous reference for normalization.

### Overexpression of NFATc1 in RAW264.7 Cells

Lentivirus preparation and infection were performed as previously described (Chen et al., 2017a). Briefly, whole mouse RNA was isolated and amplified by polymerase chain reaction (PCR) with NFATc1 primers, and the products were cloned into the pcDHGFP lentivector (CD511A-1; System Biosciences, Palo Alto, CA, United States) to construct the pcDH-NFATc1 expression vector. The 293 T-cell line was transfected with plasmids of the recombinant vectors to produce Lv-NFATc1. RAW264.7 cell suspension was then seeded into 6-well plates and Lv-NFATc1 was added at a multiplicity of infection of 20, followed by culturing for 5 days. After 18β-GA was added, osteoclastogenesis was determined using TRAP staining.

### Co-immunoprecipitation (Co-IP)

Co-immunoprecipitation (Co-IP) was performed as previously described (Liu et al., 2016). RAW264.7 cells treated with or without RANKL in the presence or absence of 18β-GA were digested using cell lysis buffer and centrifuged for 30 min followed by collection of the supernatant. TRAF6 and TRAF6-specific IgG were added to the supernatant and the mixture was incubated for 12 h. IgG agarose beads were then added and incubated for 2–4 h, followed by centrifugation. The IgG agarose beads were isolated and washed with PBS, and the cleanout buffer was collected and resolved by SDS-PAGE, followed by western blot analysis.

### PCR Analyses

Briefly, TRIzol reagent (Invitrogen, Carlsbad, CA, United States) was used to extract whole cell RNA as previously described (Koide et al., 2009). PCR primers used were mouse NFATc1, ATGACGGGGCTGGAGCAGGA (forward), and TTAGGAGTGGGGGGATCGTGC (reverse); mouse RANK, 5′-CTGCTCCTCTTCATCTCTGTG-3′ (forward) and 5′-CTTCTGGAACCATCTTCTCCTC-3′ (reverse); and mouse c-fms, 5′-TTCACTCCGGTGGTGGTGGCCTGT-3′ (forward) and 5′-GTTGAGTAGGTCTCCATAGCAGCA-3′ (reverse).

### *In Vivo* Experiments

Eight-week-old C57BL/6 female mice were obtained from Shanghai Slack (Shanghai, China). The feeding, maintenance, and use of these animals were in accordance with the principles of the Ethics Committee on Animal Experiments of the Changhai Hospital (SYXK 2015-0017). All animal experiments were performed under pathogen-free conditions. The mice were randomly divided into three groups of six animals using Excel software (Microsoft, Redmond, WA, United States), each according to their weights. After ovariectomy, the three groups were classified as the sham-operated group, the ovariectomized (OVX) treatment with vehicle group, and the ovariectomy and 18β-GA treatment group. After pre-experiment, we confirmed that the maximum concentration of 18β-GA in mice was 50 mg/kg. The process of ovariectomy has been previously described (Qiu et al., 2007). The mice in OVX and treatment group were injected intraperitoneally (i.p.) with vehicle and 18β-GA (50 mg/kg) every day. After 6 weeks, all mice were anesthetized with chloral hydrate. Then, the femur and arterial blood were collected.

### Bone Histomorphometrical Analyses

Bone histomorphometrical analyses were performed to evaluate the number of trabecular bone and osteoclasts in femoral metaphysis as previously described (Koide et al., 2009). Briefly, femurs were fixed and decalcified for 2 weeks. Sections (4-μm thickness) were then prepared with a microtome and stained with hematoxylin and eosin (HE), then TRAP staining was used to visualize the osteoclasts.

### Microcomputed Tomography Analyses

Microcomputed tomography (Skyscan, Antwerp, Belgium) was used to analyze the trabecula of the femur. The analyses were performed using 80 kv, 124 μA, and 8 μm resolution. Structural parameters for the metaphyseal region of the proximal tibiae were analyzed with the built-in software using the bone mineral density (BMD), bone volume/total volume (BV/TV), bone surface area/total volume (BS/TV), and trabecular number (Tb.N).

### Serum Biochemistry

Under anesthesia, the right eye ball of each mouse was removed and the blood was collected via fundus venous plexus. The serum was then collected and centrifuged. The levels of IL-6, CTX-1, TNF-α, and TRAcp5b in the serum were measured using ELISA kits (Anogen, Mississauga, ON, Canada) following the manufacturer’s instructions.

### Calcein Double-Labeling Analyses

Mice were injected with calcein (10 μg/g) twice at 3 days and 10 days before euthanasia. The femurs were collected and the sections were prepared with EXAKT 300CP without decalcification. Fluorescence images were then obtained using a microscope with 100× magnification (BX53, Olympus, Tokyo, Japan).

### Statistical Analyses

Data were expressed as the mean ± SD. One-way analysis of variance was used to compare ≥3 groups, and the two-independent sample Student’s *t*-test was used for comparisons between two groups using SPSS statistical software for Windows, version 21.0 software (IBM, Armonk, NY, United States). A value of *p* < 0.05 was considered statistically significant.

## Results

### Treatment With 18β-GA Had no Significant Effects on Osteogenic and Adipogenic Differentiation of Bone Marrow-Derived Mesenchymal Stem Cells (BMSCs)

To investigate the effects of 18β-GA on osteogenesis and adipogenic differentiation of BMSCs, we performed ALP, Oil Red O, and Alizarin Red staining. ALP- and Alizarin Red-positive cells increased after induction with or without 18β-GA, showing osteogenesis differentiation was not affected by 18β-GA. Oil Red O staining showed that 18β-GA had no significant effects on adipogenic differentiation (**Supplementary Figure S1**). These results showed that 18β-GA had no significant effects on adipogenic differentiation and osteogenesis of BMSCs.

### Treatment With 18β-GA Suppressed Osteoclastogenesis *in Vitro*

We performed an MTT assay before the *in vitro* study. The results showed that 18β-GA was not cytotoxic below 27.81 μg/ml (**Figure 1B**). To explore the effects of 18β-GA on osteoclastogenesis, we used two standard cellular osteoclastogenesis models involving BMMs and RAW264.7 cells. After induction with M-CSF and RANKL, the cells were treated with 6.9525, 13.905, and 27.81 μg/ml 18β-GA. After 7 days, the number of TRAP-positive cells was significantly increased in the control group, and 18β-GA significantly inhibited osteoclastogenesis in a dose-dependent manner (**Figures 1C,D**).

### Treatment With 18β-GA Inhibited Osteoclasts Functions *in Vitro*

**FIGURE 2:**
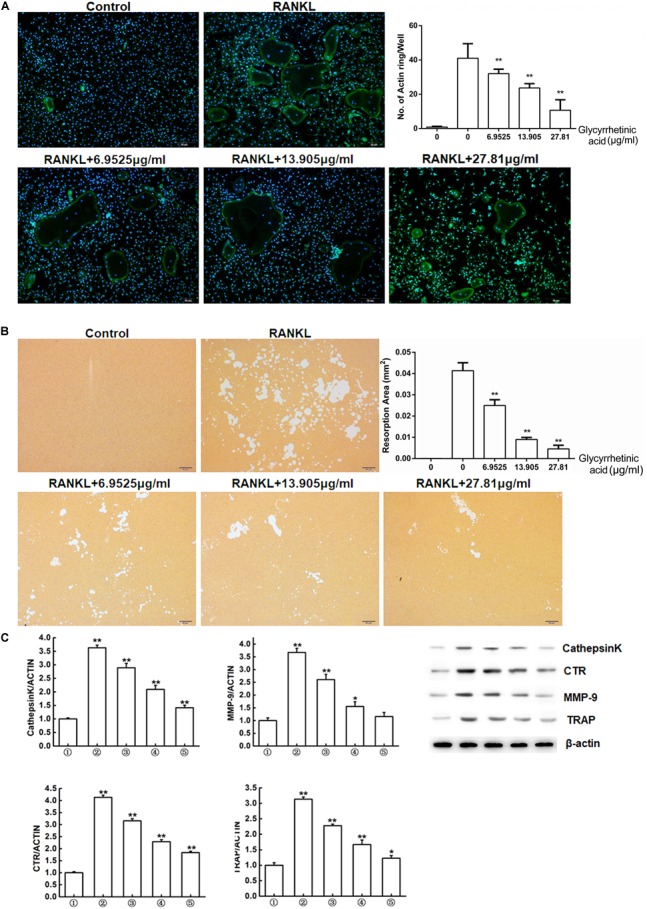
18β-GA inhibits osteoclasts function *in vitro*. **(A)** Actin ring structures of osteoclasts and quantification of the actin ring. **(B)** Pits formation assay of osteoclasts and quantification of pits area. 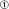 RAW264.7 cells; 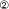 RAW264.7 cells induced with M-CSF, RANKL, and PBS; 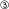 RAW264.7 cells induced with M-CSF, RANKL and treated with 6.9525 μg/ml 18β-GA; 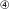 RAW264.7 cells induced with M-CSF, RANKL and treated with 13.905 μg/ml 18β-GA; 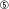 RAW264.7 cells induced with M-CSF, RANKL and treated with 27.81 μg/ml 18β-GA. **(C)** Western blot and optical density analysis of the expression of Cathepsin K, CTR, MMP-9, and TRAP with β-actin as reference (^∗^*P* < 0.05, ^∗∗^*P* < 0.01 versus 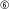).

To examine the effects of 18β-GA treatment on the bone resorption function of osteoclasts, we characterized actin ring formation, which is a prerequisite for bone resorption. 18β-GA treatment significantly reduced the size and number of the actin rings in a dose-dependent manner (**Figure 2A**).

We next used the pit formation assay to evaluate whether 18β-GA treatment affected resorption. Obvious pits formed in the plates seeded with BMMs stimulated by RANKL and M-CSF, but the pit forming activity was inhibited after cells were treated with 6.9525, 13.905, and 27.81 μg/ml 18β-GA, indicating that 18β-GA inhibited the bone resorption function of osteoclasts (**Figure 2B**).

Western blot analysis showed that expression levels of osteoclastogenesis-related markers, including cathepsin K, CTR, MMP-9, and TRAP were significantly increased while treatment with 18β-GA significantly suppressed their expression (**Figure 2C**).

### Treatment With 18β-GA Inhibited the Early Stage of Osteoclastogenesis

**FIGURE 3:**
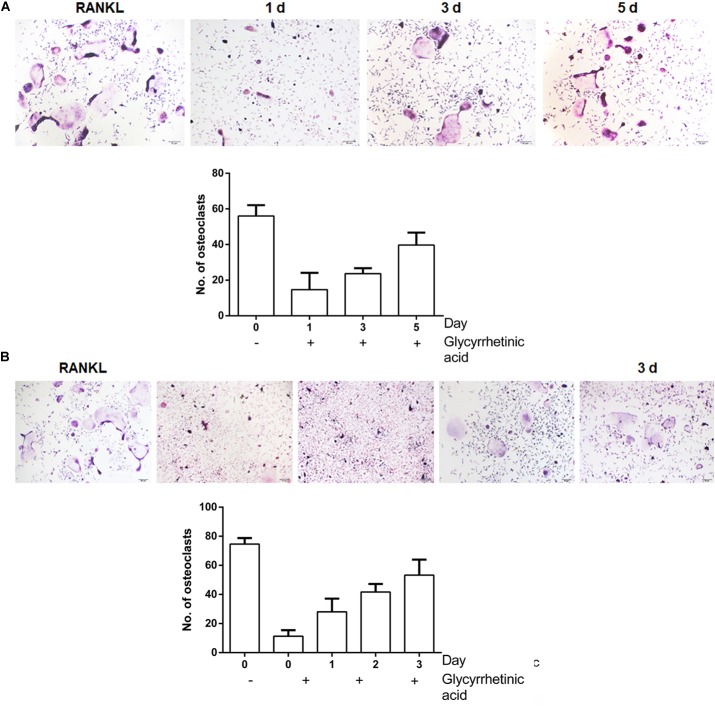
18β-GA inhibits RANKL-induced osteoclast formation at the early stage. **(A)** Effect of 18β-GA on RANKL-induced primary osteoclast precursor differentiation at different stage. **(B)** Effect of 18β-GA on RANKL-induced RAW264.7 cell differentiation at different stages.

To identify the stage at which 18β-GA treatment affected osteoclastogenesis, after induction, BMMs were treated with 18β-GA from day 0 to day 5 (**Figure 3A**) and RAW264.7 cells were treated from day 0 to day 3 (**Figure 3B**). The 18β-GA treatment inhibited osteoclast differentiation on the first day, but less effectively at later stages. Taken together, the results showed that 18β-GA exerted inhibitory effects on RANKL-mediated osteoclast differentiation at an early stage.

### Treatment With 18β-GA Inhibited RANKL-Mediated NF-κB Activation

**FIGURE 4:**
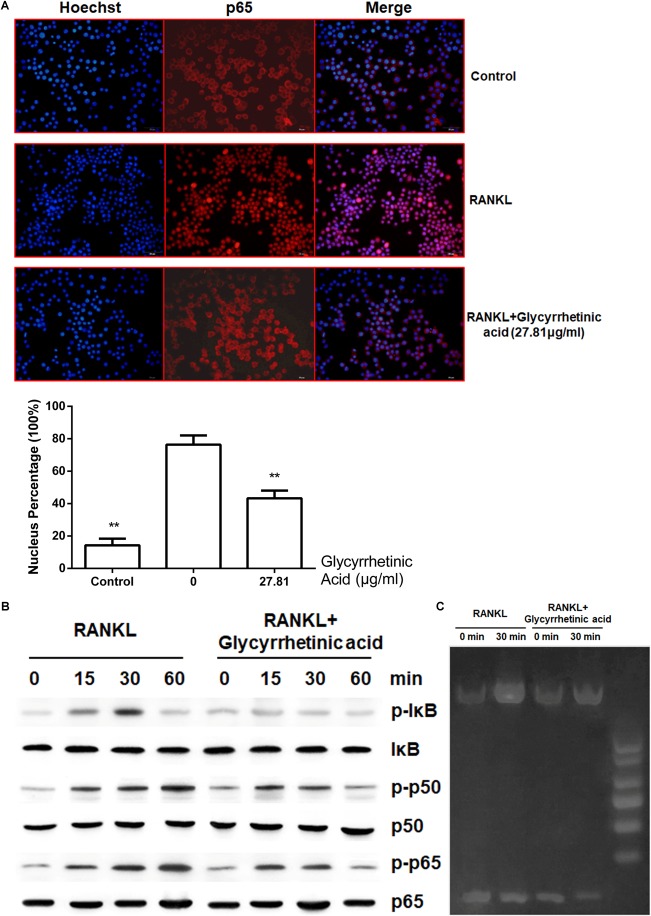
18β-GA inhibits RANKL-induced activation of NF-κB pathway. **(A)** 18β-GA inhibits RANKL-induced P65 nuclear translocation. **(B)** Phosphorylation of P65, P50, and IκB protein, which were associated with the NF-κB pathway. **(C)** The DNA-binding activity of NF-κB.

The activation of the NF-κB pathway mediated by RANKL is essential for osteoclastogenesis. To determine whether 18β-GA treatment influenced osteoclastogenesis by regulating the NF-κB pathway, we performed immunofluorescence staining of p65. In the absence of RANKL, immunofluorescence staining showed that p65 was mainly located in the cytoplasm. After stimulation by RANKL, p65 was phosphorylated and then translocated to the nucleus. However, the activation of p65 was blocked after 18β-GA treatment (*p* < 0.05) (**Figure 4A**). Western blotting showed that 18β-GA treatment inhibited RANKL-mediated phosphorylation of IκB, p65, and p50 (**Figure 4B**). We then used an EMSA to detect the DNA binding activity of NF-κB. The results showed that 18β-GA treatment decreased the DNA binding activity of NF-κB after RANKL stimulation (**Figure 4C**).

### Treatment With 18β-GA Inhibited RANKL-Mediated Activation of the MAPK Pathway

**FIGURE 5:**
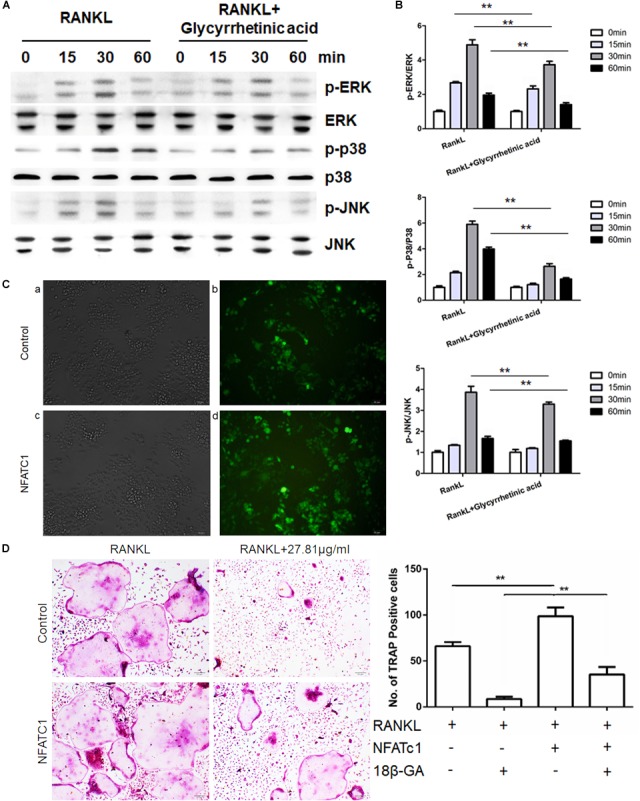
18β-GA inhibits RANKL-induced activation of the MAPK pathway. **(A,B)** Phosphorylation of ERK, P38, and JNK, which was associated with the MAPK pathway. **(C)** Fluorescence of cells infected with lentivirus after 72 h. **a, b** was infected with Lv-shRNA-NFATC1 and **c, d** was infected with Lv-NFATc1. **(D)** Overexpression of NFATc1 could reverse the effect of 18β-GA in osteoclastogenesis.

To evaluate the effects of 18β-GA treatment on the MAPK pathway, we characterized the phosphorylation of p38, JNK, and ERK, which are the three major subfamilies of MAPK (**Figures 5A,B**), by western blotting. The results showed that p38, ERK, and JNK were significantly phosphorylated after RANKL stimulation, and 18β-GA treatment significantly inhibited their activation. These results showed that 18β-GA treatment inhibited RANKL-mediated MAPK pathway activation.

### Overexpression of NFATc1 Reversed the 18β-GA Inhibitory Effects on Osteoclastogenesis

To determine where 18β-GA exerted its effects, we overexpressed NFATc1 in RAW264.7 cells and induced osteoclastogenesis (**Figure 5C**). When NFATc1 was overexpressed, the number of osteoclasts was significantly increased. Treatment with 18β-GA almost completely blocked osteoclastogenesis, while the inhibitory effects of 18β-GA were reversed after NFATc1 was overexpressed (**Figure 5D**). These results showed that overexpression of NFATc1 reversed the inhibitory effects of 18β-GA.

### Treatment With 18β-GA Blocked RANK–TRAF6 Association and Suppressed M-CSF-Induced RANK Expression

**FIGURE 6:**
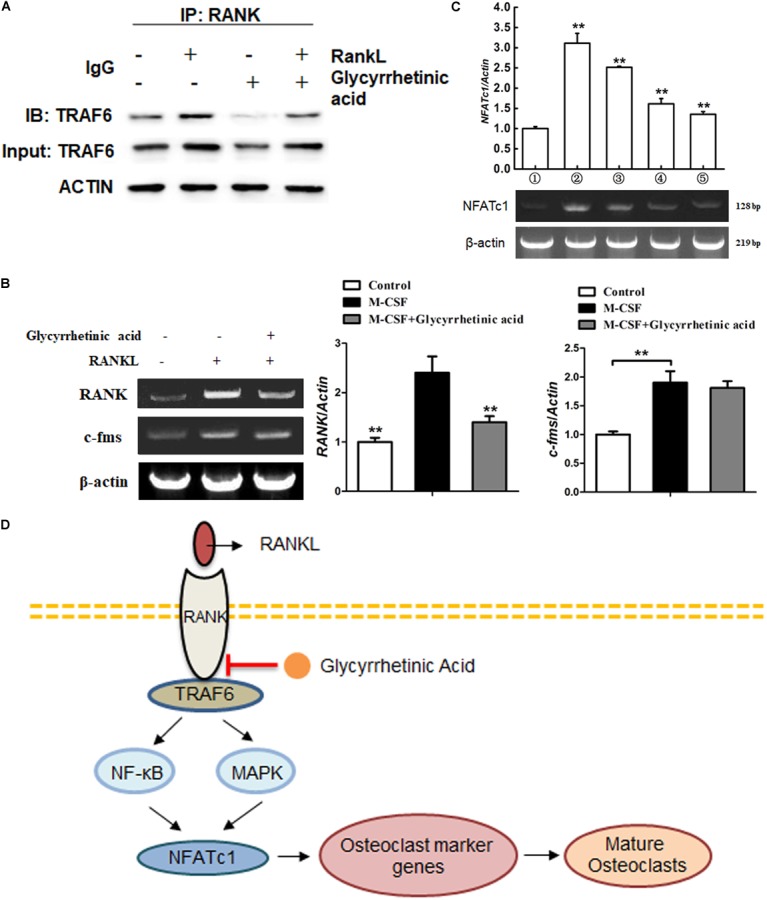
18β-GA inhibits the activation of NFATc1 on osteoclastogenesis. **(A)** 18β-GA suppresses the binding of RANK and TRAF6. **(B)** 18β-GA had no significant effects on the expression of c-Fms mediated by M-CSF. However, the RANK expression was decreased by 18β-GA. **(C)** 18β-GA inhibits the mRNA levels of NFATc1. **(D)** 18β-GA inhibits osteoclastogenesis through NF-κB pathway and MAPK pathway of RANKL signaling.

We performed Co-IP to identify the role of 18β-GA in the recruitment of TRAF6 after RANK was activated (**Figure 6A**). The results showed that RANKL combined with RANK induced the recruitment of TRAF6. Treatment with 18β-GA significantly inhibited the binding of RANK and TRAF6, indicating that 18β-GA blocked RANKL-induced endogenous RANK recruitment of TRAF6. To investigate the effects of 18β-GA on BMM proliferation and transition to preosteoclasts, we evaluated the expression of RANK and c-Fms by end-point -PCR after induction with M-CSF. The results showed that 18β-GA treatment had no significant effects on the expression of c-Fms mediated by M-CSF. RANK expression was decreased by 18β-GA treatment, indicating that 18β-GA suppressed the RANK expression of BMMs (**Figure 6B**). RT-PCR showed that NFATc1 was upregulated during osteoclastogenesis, while treatment with 18β-GA inhibited its upregulation (**Figure 6C**). These results showed that treatment with 18β-GA blocked the recruitment of TRAF6 after RANK was activated by RANKL and inhibited NFATc1 transcription by inhibiting the NF-κB and MAPK pathways (**Figure 6D**).

### Treatment With 18β-GA Reduced Bone Loss in OVX Mice

**FIGURE 7:**
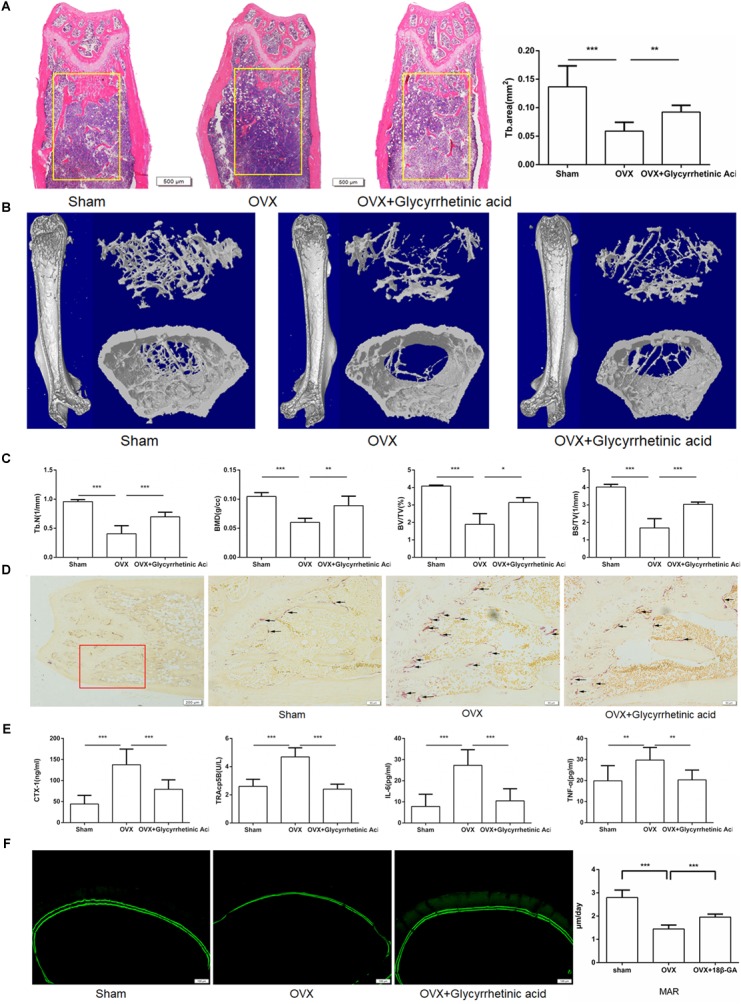
18β-GA ameliorates ovariectomy-induced bone loss *in vivo*. **(A)** Representative HE staining of distal femoral sections and quantification of trabecular area from each group 6 weeks after the operation. **(B)** Micro CT analysis of the distal femur from sham, OVX, and OVX + 18β-GA group. **(C)** Calculations of trabecular number (Tb.N), bone mineral density (BMD), bone surface area/total value (BS/TV) and bone value/total value (BV/TV). **(D)** Representative TRAP-stained histologic distal femur sections from sham, OVX and OVX + 18β-GA group. **(E)** Serum IL-6, TNF-α, TRAcp5b, and CTX-1 were examined. **(F)** Calcein double labeling with a 7-day interval (Scale bar: 100 μm). Mineral apposition rate (MAR) is significantly decreased in OVX mice and reversed by 18β-GA (^∗^*P* < 0.05, ^∗∗^*P* < 0.01, ^∗∗∗^*P* < 0.001).

To further determine the effects of 18β-GA *in vivo*, we used an ovariectomized mouse model, which mimicked postmenopausal osteoporosis. As shown by HE staining, intraperitoneal injection of 18β-GA (50 mg/kg) reduced trabecular bone loss in the distal femur (**Figure 7A**). These findings were consistent with the microcomputed tomography results as shown by Tb.N, BMD, BV/TV, and BS/TV, and the 2- and 3-dimensional structures (**Figures 7B,C**). To further investigate the effects of 18β-GA on osteoclastogenesis, we performed TRAP staining. The results showed that 18β-GA significantly reduced the number of TRAP-positive cells around the trabecula, which indicated that osteoclastogenesis was inhibited by 18β-GA treatment (**Figure 7D**). Osteoclast activity and inflammation status were also investigated by determining the serum levels of TRAP5b, crosslinked C-telopeptide 1 (CTX-1), TNF-α, and IL-6. Compared to the OVX group, TRAP5b, CTX-1, TNF-α, and IL-6 were significantly reduced by treatment with 18β-GA (**Figure 7E**). We then performed calcein double-labeling analyses to determine whether 18β-GA treatment affected the mineralization of bone matrix. The mineralization apposition rate was significantly decreased in the OVX mice and reversed by 18β-GA treatment, suggesting that treatment with 18β-GA increased the mineralization of the bone matrix (**Figure 7F**). These results showed that 18β-GA reduced bone loss in OVX mice by inhibiting osteoclastogenesis.

## Discussion

In this study, we showed for the first time that 18β-GA inhibited osteoclastogenesis, actin formation and osteoclasts functions *in vitro* while had no significant effects on osteogenesis. As for the molecular mechanisms, 18β-GA inhibited RANKL-induced activation of NF-κB and MAPK signaling pathways at the early stage. 18β-GA decreased RANK expressions induced by M-CSF and blocked the recruitment of TRAF6 after RANK was activated by RANKL. We further showed that 18β-GA protected OVX-induced bone loss, significantly increased mineral apposition rate in OVX mice *in vivo.*

Bone is constantly changing through bone formation by osteoblasts and resorption by osteoclasts. Excessive bone resorption by over-activated osteoclasts leads to a number of pathological circumstances like postmenopausal osteoporosis, RA, etc. Natural products are an abundant resource of therapeutic candidates. It has been demonstrated that many nature products exact are helpful for osteopenic diseases. Nevertheless, many still remain unknown. 18β-GA, extracted from liquorice, is a promising bioactive component with anti-inflammatory and antioxidant properties. Recent studies have mainly focused on its immunoregulatory activities. It prevented free fatty acid-induced hepatotoxicity by stabilizing the integrity of lysosomes and mitochondria (Wu et al., 2008), decreased CCl_4_-induced liver damage by inhibiting the NF-κB pathway (Cao et al., 2017), and attenuated airway inflammation in the asthmatic mouse model (Kim et al., 2017). Because the imbalance between osteoclasts and osteoblasts is regarded as a disruption of immune balance, we deduced that 18β-GA could be protective against OVX-induced bone loss, which has not been previously reported.

In this study, we first performed MTT assays and selected 18β-GA concentrations of 6.9525, 13.905, and 27.81 μg/ml for *in vitro* studies to exclude the cytotoxic effects of 18β-GA. 18β-GA treatment had no significant effects on BMSC osteogenesis and adipogenesis (Li et al., 2011). It has been well established that M-CSF initiates osteoclast precursors proliferation and differentiation into osteoclasts (Rizzoli et al., 2011; Liao et al., 2013). In our study, we found that 18β-GA had little effect on osteoclast precursor proliferation, significantly inhibited RANK expression which subsequently induces differentiation of osteoclast precursors into osteoclasts. After RANKL binding to RANK on preosteoclasts, TRAFs were recruited, which led to the activation of the NF-κB and MAPK pathways (Shimizu et al., 2016; Zeng et al., 2017). Treatment with 18β-GA inhibited RANKL-mediated phosphorylation of p65, p50, and IκB in the NF-κB pathway and ERK, JNK and p38 in the MAPK pathway. The most potent osteoclastogenesis transcription factor, NFATc1, was also significantly inhibited by 18β-GA treatment by RANKL stimulation. Thus, 18β-GA treatment blocked the transition from preosteoclasts to osteoclasts in the presence of RANKL.

We then determined the stage when 18β-GA treatment functioned. The results showed that 18β-GA treatment mainly inhibited RANKL-induced osteoclastogenesis at the early stage from preosteoclasts to mature osteoclasts. NFATc1, which is responsible for the expression of various osteoclastogenesis-related genes, is an important regulator of osteoclast function and differentiation. RT-PCR showed that 18β-GA suppressed RANKL-induced NFATc1 transcription. To determine where 18β-GA affected osteoclasts differentiation, we overexpressed NFATc1 in RAW264.7 cells and found that the inhibitory effects of 18β-GA on osteoclastogenesis were partially compromised, suggesting that 18β-GA was active in the upstream portions of pathways. We then performed Co-IP and found that 18β-GA blocked the interactions of RANK and TRAF6.

We used MMP-9, TRAP, CTR, and cathepsin K as osteoclastogenesis-related markers. MMP-9, expressed by osteoclasts, is a member of the matrix metalloproteinases family, which degrades the organic matrix of bones (Nyman et al., 2011) and is an indicator of osteoclastogenesis in PMOP (Paiva and Granjeiro, 2017). Using TRAP staining, our results showed that 18β-GA treatment inhibited the expression of these markers.

Osteoporosis, an aging-related chronic disease, is characterized by progressive bone loss and a high incidence rate of fracture, and is associated with an imbalance of bone resorption and bone formation (Stralberg et al., 2013; Lentle et al., 2017). It has been widely recognized that excessive osteoclastogenesis, resulting from over activation of RANKL signaling pathways because of estrogen withdrawal, plays an important role in the pathogenesis of PMOP (Hwang and Putney, 2012). After menopause, the level of the potent inflammation inhibitor, estrogen, decreases, accompanied by increased secretion of RANKL and proinflammatory cytokines such as IL-1, IL-6, and TNF-α, which results in the overactivation of osteoclasts and an increase in bone formation. Because the balance between osteoclasts and osteoblasts is disrupted, the bone is gradually resorbed and PMOP occurs (Stoecker et al., 2017). Thus, inhibiting osteoclastogenesis remains an important strategy for PMOP treatment as well as osteoclast-related bone diseases (Wu et al., 2017).

*In vivo*, we found that 18β-GA treatment reduced bone loss in ovariectomized mice. HE staining and micro-CT analyses showed that the bone loss was significantly reduced. In addition, TRAP staining showed that positive cells were significantly reduced surrounding the trabecula in the distal femur after treatment with 18β-GA. Serum levels of IL-6, TNF-α, CTX-1, and TRAcp5b, which were elevated after OVX (Moriwaki et al., 2014; Sucur et al., 2014), were reduced after treatment with 18β-GA. These *in vivo* studies indicated that the main mechanism of action of 18β-GA involved suppressing inflammation and inhibiting osteoclastogenesis.

However, further research will be needed to further clarify the mechanism of 18β-GA inhibition of osteoclastogenesis and to determine the roles of 18β-GA in the treatment of PMOP as well as in osteoclastogenesis-related diseases.

Collectively, our study showed that 18β-GA inhibited RANKL-induced osteoclastogenesis *in vitro* and reduced ovariectomy-induced bone loss *in vivo*. For molecular mechanisms, 18β-GA inhibited M-CSF-induced RANK expression, suppressed NF-κB and MAPK signaling pathways during osteoclastogenesis, and blocked RANK–TRAF6 upstream interactions in signaling pathways. Overall, our study strongly suggested that 18β-GA could be an effective candidate for osteoclast-related pathological bone diseases including PMOP.

## Author Contributions

XC, XZ, and JS: study design. XZ and ZY: data collection. XL and LQ: data analysis. XC and JS: data interpretation. XC, XZ, and ZQ: drafting manuscript. XC, XZ, and ZQ: revising manuscript content. SJ: approving final version of manuscript.

## Conflict of Interest Statement

The authors declare that the research was conducted in the absence of any commercial or financial relationships that could be construed as a potential conflict of interest.

## Supplementary Material

FIGURE S1 18β-GA showed no significant effects on osteogenic and adipogenic differentiation of BMSCs.
