# Supplementary figures and images for: 18β-Glycyrrhetinic Acid Inhibits Osteoclastogenesis In Vivo and In Vitro by Blocking RANKL-Mediated RANK–TRAF6 Interactions and NF-κB and MAPK Signaling Pathways
Source: Front Pharmacol. 2018 Jun 20;9:647. doi: 10.3389/fphar.2018.00647 (PMC6019442; doi:10.3389/fphar.2018.00647)

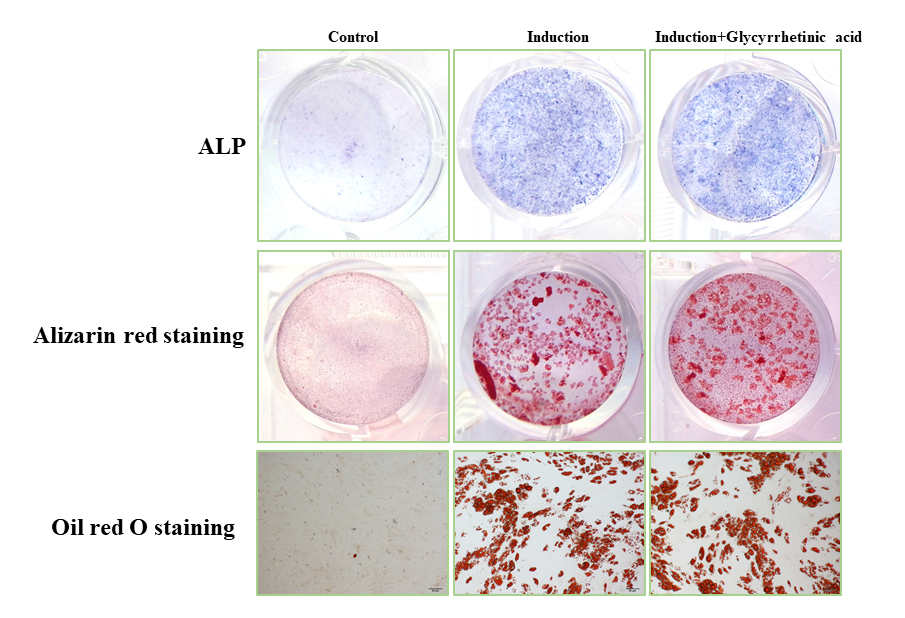

Supplement: FIGURE S1 — 18β-GA showed no significant effects on osteogenic and adipogenic differentiation of BMSCs. [file Image_1.TIF]
